# Development and validation of the German Performance-related Questionnaire for Musicians (PQM) for measuring situational music performance anxiety

**DOI:** 10.3389/fpsyg.2026.1722181

**Published:** 2026-03-03

**Authors:** Claudia Spahn, Manfred Nusseck

**Affiliations:** Freiburg Institute for Musicians’ Medicine, University of Music Freiburg, Medical Center and Faculty of Medicine – University of Freiburg, Freiburg Center for Research and Teaching in Music, Freiburg, Germany

**Keywords:** music performance anxiety, performance quality, questionnaire, self-assessment, self-efficacy, situational behavior

## Abstract

**Introduction:**

When performing in public, musicians experience varying levels of music performance anxiety (MPA). The degree of MPA is influenced by various internal and external factors and differs between performances. Previous studies have mainly focused on the general disposition of MPA, but comparatively limited attention has been given to the experience of MPA in particular performance situations. In this study, the Performance-related Questionnaire for Musicians (PQM) is introduced and validated. The questionnaire was developed to assess situational MPA, thus filling a gap in standardized questionnaires relating to individual performances.

**Methods:**

The fourth revised German version of the PQM was tested regarding the reliability of the factor structure and the validity on a sample of 605 musicians. The PQM questionnaire focuses on aspects of situational MPA referring to a just-finished performance. It needs to be completed directly after a performance and considers retrospectively the times before and during the performance, as well as the moment when filling in the questionnaire after the performance. For the analysis, exploratory factor analysis (EFA) and confirmatory factor analysis (CFA) were performed. Furthermore, the questionnaire was implemented in a mobile application for the individual acquisition of PQM results across various performances. In a case study, data were analysed from 31 performances of one musician.

**Results:**

A three-dimensional factor structure at the time points before, during, and after the performance showed reliable consistencies in the EFA, and the CFA confirmed the structure with adequate model fit statistics. The three dimensions represent, first, the degree of MPA symptoms (the higher the scale, the more severe the MPA); second, coping with MPA (the higher the scale, the more positive the coping); and third, self-efficacy (the higher the scale, the more positive the self-efficacy). The results of the case study with the mobile application showed individual differences and consistencies in situational MPA between performances.

**Discussion:**

The results show that the PQM is a valid tool for assessing situational MPA. The implementation as a mobile application is described as very practical and supports the use of the PQM for individual self-assessment and feedback.

## 1 Introduction

For musicians, performing in front of an audience is a central aspect of professional musical practice. Many artists experience specific emotional, physical, and cognitive responses to performance situations, such as rehearsals, auditions, concerts, and productions, which are characterized by anxiety and arousal. This phenomenon is referred to as music performance anxiety (MPA) (Kenny, 2011; Spahn, 2012, 2015; Osborne and Kirsner, 2022). Effective management of MPA is crucial for musicians to optimize performance quality and support a successful musical career (Barros et al., 2022; Blair and Van Der Sluis, 2022; Wang and Yang, 2024).

MPA can have both debilitating and facilitating effects on musical performance (Herman and Clark, 2023) and is best conceptualized as a continuum of intensity (Spahn, 2012). At its extreme, MPA may manifest as a pathological disorder (Spahn, 2012; Fernholz et al., 2019). Variability in the spectrum and severity of MPA can be accounted for by stress–response models (Twitchell et al., 2025) and neurophysiological mechanisms (Spahn, 2015). The individual appraisal of performance demands has a decisive influence on whether MPA is facilitating or debilitating. A facilitative MPA occurs when resources are perceived as greater than demands and the performance is evaluated as a challenge, whereas a debilitating MPA occurs when demands are perceived as exceeding available resources, i.e., when the person feels threatened (Twitchell et al., 2025). If the performance can be regarding as a challenge and MPA in a broader mindset as beneficial, it can enhance motivation, concentration, attentional focus and performance quality, often accompanied by a sense of anticipatory enjoyment (Spahn, 2012; Kaleńska-Rodzaj, 2021).

To summarize the wide range of influencing factors on MPA (Nicholl and Abbott, 2024), Papageorgi et al. (2007) suggest three categories: (1) personal characteristics such as age, gender and personality, especially predisposition to anxiety; (2) task-related factors such as preparation time, motivation, complexity of the repertoire, and the significance of the performance; and (3) contextual factors including audience size and composition, and whether the performance is solo or ensemble-based.

Personal factors influencing MPA include psychological vulnerability and prior anxiety-related experiences (Osborne and Kirsner, 2022). MPA may also co-occur with other anxiety disorders, such as social anxiety disorder (Nicholl and Abbott, 2024) or reflect broader trait anxiety (Kenny, 2011, 2023; Spahn, 2012). Given that anxiety is the central emotional component of MPA, excessive levels can result in loss of control, feelings of helplessness, and a heightened sense of threat (Riemann, 2008; Spahn, 2012).

The foundational model developed by Yerkes and Dodson (1908) has been widely adopted in MPA research, describing the relationship between arousal and performance quality as an inverted U-shape. However, a key limitation of the model is its unidimensional approach to stress arousal (or activation of the sympathetic nervous system), which treats stress as a uniform concept without distinguishing between different types of stress responses (Twitchell et al., 2025). Twitchell et al. (2025) therefore proposes reconceptualizing the U-shape model for MPA along the more differentiated perspective of the biopsychosocial model, which encompasses qualitative components of MPA. It could also be taken into account that the subjective assessment of the difficulty of a task is the actual decisive factor in whether stress promotes or impairs performance, rather than simply the degree of arousal (Twitchell et al., 2025).

Contextual factors such as the performance setting—solo or ensemble—or the subjective perception of the performance situation influence the severity of MPA. Casanova et al. (2018) found that university music students reported higher levels of MPA during solo performances compared to group performances, and during concerts compared to rehearsals. In contrast, Murphy et al. (2025) found that nearly half of the musicians experienced MPA as beneficial during solo performances. Performance contexts in which MPA was perceived as supportive were significantly associated with positive emotions (Kaleńska-Rodzaj, 2023; Murphy et al., 2025).

One of the most influential determinants of a successful musical performance is self-efficacy (Zelenak, 2024). Rooted in Bandura’s (1997) theoretical framework, self-efficacy is defined as “the belief in one’s own capabilities to organize and execute the courses of action required to produce a given attainment”. Numerous studies have demonstrated that higher self-efficacy is associated with more active use of strategies to cope with MPA and consistently correlates negatively with MPA (McCormick and McPherson, 2003; McPherson and McCormick, 2006; Hewitt, 2015; Miksza, 2015; Bugos et al., 2016; González et al., 2018). More recently, Wang and Yang (2024) investigated the interrelationships between MPA, self-efficacy, and future career expectations among Chinese music students. Their findings indicate that MPA is negatively associated with self-efficacy. Additionally, self-efficacy acts as a partial moderator between MPA and career expectations, suggesting that strengthening students’ self-efficacy may enhance their professional aspirations and mitigate the adverse effects of MPA.

Studies on coping strategies for MPA have shown that effective management particularly involves the cognitive regulation of catastrophic thoughts and the enhancement of positive emotional states (Osborne and Kirsner, 2022; Niering et al., 2023). Regarding the performance situation itself, different phases—before, during, and after—are characterized by distinct levels of MPA (Papageorgi et al., 2007). In some studies, MPA tends to be highest immediately before a performance (Spahn, 2012). Comparing MPA 1 h before the performance, immediately before the performance and during the performance, Papageorgi et al. (2013) found that MPA levels peaked immediately before the performance and decreased subsequently during the performance. In contrast, Studer et al. (2014) demonstrated that symptoms of music performance anxiety can peak during the performance itself. Using respiratory monitoring as an important dimension to understanding music performance situations and MPA, Guyon et al. (2020) suggest that stability in the respiratory time parameters decreased during the post-performance phase compared to the pre-performance phase of a public session. In a study with university music students, Gomez et al. (2018) showed even prolonged neuroendocrine effects on post-concert days after solo music performances. All in all, the systematic assessment of dynamic changes in MPA throughout the entire course of a performance seems ambiguous.

Existing self-assessment questionnaires that measure the overall level of MPA (Gómez-López and Sánchez-Cabrero, 2023), such as the Performance Anxiety Inventory (PAI) (Nagel et al., 1981), the Kenny Music Performance Anxiety Inventory (K-MPAI) (Kenny, 2011, 2023), or the Performance Anxiety Inventory for Musicians (PerfAIM) (Barbeau, 2011), as well as the Performance Anxiety Questionnaire (PAQ) (Cox and Kenardy, 1993), primarily assess MPA from a general perspective and conceptualize it largely as dispositional anxiety (Kenny, 2011). These questionnaires can be administered at any time and typically rely on a retrospective self-assessment of individual performance experience in general.

Questionnaires assessing dispositional MPA have been widely employed in epidemiological and intervention studies examining the prevalence and severity of MPA. Recent reviews have come to inconsistent conclusions regarding the effectiveness of interventions for managing MPA. In addition to methodological limitations such as inadequately controlled study designs and low effect sizes, these reviews also highlight a central conceptual issue: intervention outcomes typically refer to reductions in general MPA and fail to capture situation-specific dynamics or changes in MPA across the temporal course of a performance (Barros et al., 2022; Blair and Van Der Sluis, 2022; Niering et al., 2023).

In this context, the Performance-specific Questionnaire for Musicians (PQM) presented in this study was developed to address the gap in assessing temporal variations of MPA throughout a performance. The questionnaire was designed to be completed shortly after a performance and retrospectively addresses the time points immediately before the performance and during the performance, as well as the actual time when the performance has ended. This approach enables the investigation of how musicians manage MPA within a particular performance situation. In contrast to the assessment of dispositional MPA at any time, the PQM refers to a specific performance and captures state-related MPA. It is therefore more sensitive to situational factors and allows for the determination of direct influences on the management of MPA across the performance. By using the PQM and receiving performance-related feedback, individuals can also be prevented from generalizing a negative experience in a particular performance context to all future performance contexts in a more integrated approach (Twitchell et al., 2025).

The PQM comprises three scales, i.e., the functional coping scale, the symptoms of MPA scale, and the self-efficacy scale, which are assessed at each time point of the performance. These scales were developed to reflect distinct components of MPA and to allow for the analysis of how these components change over the course of a performance. The questionnaire has already been applied in several studies (see below). The present study details its development, validation, and psychometric properties.

## 2 Performance-specific Questionnaire for Musicians (PQM)

### 2.1 Development of the PQM and previous versions

The Performance-specific Questionnaire for Musicians (PQM) was developed to assess aspects of MPA within a specific live performance situation. It was originally created for an intervention study that examined the effectiveness of performance training for music students. Unlike the students in the control group, who did not receive any intervention, the students in the intervention group participated in a seminar during one semester. Both groups underwent a simulated audition before and after the intervention period and completed the PQM questionnaire relating to the respective audition (Spahn et al., 2016). The questionnaire was designed to enable a comparative assessment of MPA for the respective auditions. At the same time, the aim was to use the questionnaire to investigate the course of MPA across the performance, referring to time points before, during, and after a performance, as suggested by Papageorgi et al. (2007). As part of the study involving music students at a German university of music, the questionnaire was initially developed in German (“Fragebogen zum Auftritt für Musiker*innen FZAM”) and contained 18 individual questions on various aspects of MPA. The questionnaire was completed directly after the musical performance, and the statements related retrospectively to the times before and during the performance, as well as to the time when filling in the questionnaire after the performance. Due to the promising results gained from using the questionnaire in the study with the music students (Spahn et al., 2016), it was further developed. It was used with a larger sample and tested for reliability and validity (Biwer, 2015).

The first revised version of the questionnaire was based on the theoretical model of the four manifestations of symptoms of MPA (Kenny, 2011; Spahn, 2012): affective symptoms such as expressions of anxiety and changing emotions; cognitive symptoms such as thoughts revolving around the performance situation, i.e., worries, self-doubt, or catastrophizing; behavioral symptoms such as agitation and avoidance; and physiological symptoms such as an increased pulse rate and breathing frequency, clammy hands, and a dry mouth.

Based on these four manifestations, a questionnaire with 49 items was designed, referring to the three time points before, during, and after the performance, using latent scales with 3–4 items for each MPA manifestation (Biwer, 2015). The validation sample for this second version of the PQM consisted of *N* = 130 musicians from various university orchestras and big bands. The average age was M = 25.5 years (SD = 5.67 years), and 53% were female while 47% were male musicians. First, the item difficulties (Bühner, 2021) were checked. After excluding those with poor item skewness (< −2 or >2), an exploratory principal component factor analysis with Varimax rotation and Kaiser normalization was performed for each time point, i.e., before, during, and after the performance. The factor analysis revealed a three-factor structure with a variance explanation of 61.9% for the time point before the performance, with reliabilities for the scales between Cronbach’s alpha 0.68 and 0.70. A two-factor solution with a variance explanation of 55.6% was found for the time point during the performance, with reliabilities between Cronbach’s alpha 0.62 and 0.85. Another three-factor structure with a variance explanation of 64.6% was found for the time point after the performance, with reliabilities between Cronbach’s alpha 0.33 and 0.62.

The results showed that the presumed scale structure based on the four manifestations of symptoms of MPA could not be confirmed. It was hypothesized that a clear distinction between the four manifestation categories would be difficult to achieve empirically. Instead, a three-dimensional structure emerged. A closer look at the respective scale items revealed clear scale content orientations. The questions on the first scale showed strongly positive attitudes towards the performance (e.g., “I was able to concentrate on the task at hand”), while the second scale showed more negative attitudes (e.g., “I thought about all the things that could go wrong”). The third scale mainly contained questions that dealt with personal beliefs and approaches to the performance (e.g., “I felt well-prepared”).

For an external validation of this version of the PQM, the STAI-S (Spielberger et al., 1970) was included in the study. The state form of the STAI questionnaire measures momentary anxiety. It was intended to test whether completing the PQM retrospectively for the situation before the performance would lead to a distorted assessment. For this reason, the STAI-S was completed twice, immediately before the performance and retrospectively after the performance. Both values were compared with each other. The three PQM scales determined by the factor analyses mentioned above at each time point in the performance were correlated with the STAI-S scales. The results showed that the PQM scales before the performance correlated more strongly (*r* = 0.44**) with the STAI-S completed before the performance than with the STAI-S completed after the performance (*r* = 0.33**). The STAI-S completed after the performance did not correlate with the PQM scales before the performance (*r* = 0.07) but did correlate with the PQM scales after the performance (*r* = 0.55**). This confirmed that the questions relating to the time before the performance can be completed reliably after the performance.

Based on the results of that study, a further preliminary study was conducted with another revised version (the third version) of the PQM (Biwer, 2015). The inappropriate items were deleted, resulting in a total of 33 items. This study aimed to test the item properties again and check the reliability of the previously identified scales. For the revised version of the questionnaire, the division of the time points, i.e., before, during, and after the performance, was retained. The three-factor structure from the previous analysis was adapted for the thematic organization of the individual scales. The sample consisted of *N* = 137 musicians with an average age of M = 27.6 years (SD = 11.2 years). Fifty-six percent were female and 44% male musicians. The participants in this study were different from those in the previous study. The results confirmed the three-factor structure of the previous PQM version at every time point. The factor analysis of the PQM scales showed explained variances between 55 and 62%. Only a few items were slightly modified for the final version of the PQM due to moderate item difficulties.

In summary, the results of the pre-analyses of previous PQM versions confirmed that the questionnaire adequately describes a differentiated assessment of the handling and influence of MPA regarding a particular performance and the times before, during, and after that performance, using a three-factor model. The final version (the fourth version) of the PQM in German and in English can be found in the Supplementary material.

### 2.2 Structure and content of the PQM

#### 2.2.1 Structure and applicability

The PQM is a self-assessment instrument focusing on relevant aspects of MPA in a particular performance. In total, the current version of the questionnaire contains 33 items with 11 items for each time point, i.e., before, during and after the performance. The statements are rated on a five-point scale between 1: “not true at all” and 5: “very true.”

Seven additional items ask about the performer’s satisfaction with their performance. The questions begin with: “When considering the musical quality of my performance, I rate the…” followed by aspects of dynamic shaping, rhythmic precision, the shape of the sound, musical expression, phrasing, intonation, and overall performance. These aspects are then rated on a six-point scale from 1: “terrible” to 6: “excellent.” The content of these areas was based on the observation criteria of Mills (1987). The seven items were found to load on a single factor, and the resulting scale was defined as the self-assessed musical performance quality scale.

In addition, questions specifically about the performance are included. Participants are asked how important the performance was for them personally. The answers are made on a four-point scale (1: “not important”; 2: “less important”; 3: “important”; 4: “very important”). Participants also rate the concert difficulty in comparison to other concerts (1: “easy”; 2: “not so easy”; 3: “rather difficult”; 4: “difficult”). Finally, participants are asked to rate how difficult the performance was for them personally. This is done on a five-point response scale (1: “too low”; 3: “just right”; 5: “too high”).

When using the questionnaire, it is important to complete it while memories of these events are still fresh and have not been influenced by other activities or feedback from other people. It is recommended to fill in the questionnaire 15–30 min after the performance. To make it even easier for musicians to complete, the questionnaire was made available as a mobile application (see below).

#### 2.2.2 Content and theoretical foundation

The current version of the PQM (fourth version) has a three-dimensional structure. The three dimensions represent: (1) the degree of MPA symptoms; (2) functional coping with MPA; and (3) performance-related self-efficacy.

- The *Symptoms of MPA scale* addresses specific indications associated with manifestations of symptoms of MPA. It asks about physical symptoms, e.g., “I could sense signs of agitation in my body”; cognitive symptoms, e.g., “I thought about everything that could go wrong”; and emotional reactions, e.g., “I was unsettled by my agitation.” The higher the scale, the more severe the MPA.
- The *Functional coping scale* focuses on positive thoughts and feelings that help to manage MPA, e.g., “I managed to control my agitation and stay calm” or “I managed not to let my excitement unsettle me.” The higher the scale, the more positive the coping.
- The *Self-efficacy scale* refers back to Bandura’s (1997) original definition of “the belief in one’s own capabilities”, considering confidence in performance, e.g., “I was looking forward to going on stage and showing what I could do” or “I felt strong inside for the upcoming performance.” The higher the scale, the more positive the self-efficacy.

The special feature of the PQM is that the scales were developed in such a way that they are linked across the different time points during the performance, i.e., before, during, and after the performance. An attempt was made to make the items of the scales as similar as possible at the three points in time. However, as these require different perspectives, it was not possible to use identical items. Table 1 lists all items according to the PQM scales that were used at each point in time.

**Table 1:** List of all PQM items in each scale and their connections across the performance.

PQM Scale	A few minutes before the performance…	During the performance…	After the performance…
Functional coping scale	1 … I could concentrate on the work at hand.	16 … I could concentrate on my musical performance.	23 … I think my performance came across well.
	4 … I managed to control my agitation and stay calm.	15 … I managed to control my agitation and stay calm.	26 … I accept my performance.
	8 … I could control my agitation.	22 … I could control my agitation.	30 … I am happy about my achievement.
Symptoms of MPA scale	2 … I could sense signs of agitation in my body.	13 … I could feel the agitation in my body.	31 … I feel stressed, unhappy.
	6 … I felt limited or disabled due to the way my body reacted to my agitation.	17 … I felt limited or disabled due to the way my body reacted to my agitation.	33 … I’m mainly thinking about what did not work.
	9 … I thought about all the things that could go wrong.	12 … I could not stop thinking about what might go wrong.	28 … It’s hard to imagine that the audience liked my performance.
	10 … My agitation made me unsure.	19 … My agitation made me unsure.	24 … It’s hard to accept my performance as it was.
Self-efficacy scale	3 … I felt inner strength for the performance I was about to do.	21 … I felt inner strength.	29 … I feel inner strength for the next performance.
	5 … I could imagine the audience enjoying my performance.	18 … I had the feeling that my performance came across well.	–
	7 … I was looking forward to going on stage and showing what I could do.	14 … I had fun and wanted to show what I could do.	25 … I’m looking forward to my next performance.
	11 … I felt well prepared.	20 … I knew exactly what I was doing.	32 … This performance proves that performing suits me.

At the time points before and during the performance, the same wording was used in some items, i.e., items 4 and 15, 8 and 22, 6 and 17, as well as 10 and 19. Other items before the performance focused on the performance that lay ahead. The related items during the performance considered the actual performance, i.e., items 1 and 16, 2 and 13, 9 and 12, as well as 3 and 21. Some items required more rephrasing to address similar content, i.e., 5–18, 7–14, and 11–20. Overall, the focus shifts from pre-performance excitement and anticipation of the performance to experiencing the situation on stage during the performance.

The items relating to the situation after the performance differ from those before and during the performance in that they are in the present tense. They address the immediate state of mind after the performance, which is influenced by the experience of the preceding performance. While before and during the performance the focus is on the performance itself (items 1 and 16), after the performance a more reflective perspective on how the performance went naturally comes to the fore (item 23).

On the symptoms of MPA scale, the items before the performance related to anxiety (items 2 and 13, as well as 10 and 18) differ from the description of the current feeling in the items after the performance (items 31 and 24, respectively). In the self-efficacy scale, performers reported a feeling of inner strength and anticipation immediately before the performance, and had thoughts about the next performance just after the performance (items 3 and 21–29).

This content structure of the questionnaire reflects the fact that MPA is viewed in relation to coping and self-efficacy. The interpretation of the questionnaire scores therefore focuses on the relationship between the three scales. It is assumed that a positive approach to MPA symptoms and a high level of self-efficacy are associated with a satisfactory course of the performance.

#### 2.2.3 Studies with the current version of the PQM

In previous studies, the PQM (fourth version) has proved to be a reliable instrument for measuring situational MPA. In a recent study, a Polish translation of the PQM has been validated (Kaleńska-Rodzaj et al., 2025).

A large sample of *N* = 532 musicians has been collected and analysed (Spahn et al., 2021a). In this sample, 26% were instrumentalists in professional orchestras, 46% were instrumentalists in semi-professional orchestras, and 28% were singers in semi-professional choirs. The average age was M = 31.8 years (SD = 14.3), with 57% female and 43% male musicians. The PQM scales showed good internal reliability for the functional coping scales with Cronbach’s *α* = 0.74, the symptoms of MPA scales with Cronbach’s *α* = 0.77, and the self-efficacy scales with Cronbach’s *α* = 0.73. The self-assessment of musical quality of the performance showed a Cronbach’s *α* of 0.88.

Using this sample, a cluster analysis was performed on the PQM scales. The analysis yielded three different MPA types (Spahn et al., 2021a). In the first MPA type, the musicians exhibited low values on the symptoms of MPA scale, accompanied by high values on the functional coping and self-efficacy scales. The scale levels remained stable across the performance. Musicians in this MPA type were considered to have a healthy and robust approach to their MPA, comprising approximately 50% of the sample. In the second MPA type, around 27% of the musicians showed relatively high values on the symptoms of MPA scale before the performance but also exhibited high values on the functional coping and self-efficacy scales. The values of the symptoms of MPA scale decreased to a low level after the performance, while the other scale values remained high. These musicians seemed able to manage their MPA well and reported a positive experience after the performance, despite feeling quite nervous beforehand. The remaining musicians (23%) fell into the third MPA type. They had moderate values on the symptoms of MPA scale before the performance, but the values of the self-efficacy and functional coping scales were also relatively low. In this group, the values of the symptoms of MPA scale even increased after the performance, indicating a struggle to manage their MPA in a healthy manner. In summary, the analysis of the MPA types found that the self-efficacy scale was a strong moderating factor in handling situational MPA. The results showed the importance of self-efficacy and functional coping in supporting a positive performance experience.

In another study, the PQM scales were correlated with the Flow Short Scale (Rheinberg et al., 2003) to investigate the relationship between flow experiences and situational MPA (Spahn et al., 2021b). A subsample (*N* = 363; only orchestral musicians) from the above-mentioned large sample (Spahn et al., 2021a), in which the musicians had also completed the Flow questionnaire, was used. The symptoms of MPA scale was negatively correlated with the Flow scale, while both the functional coping and self-efficacy scales were positively associated with Flow.

An analysis of the relation between personality traits and situational MPA was considered in another study (Spahn et al., 2024). A different subsample of the previous study (Spahn et al., 2021a), in which *N* = 393 participants also completed the NEO-Five-Factor Inventory (Körner et al., 2002), was included. Using canonical correlation analysis, the results showed that personality traits were correlated with the PQM scales after the performance, but less so with the PQM scales before and during the performance. Personality characteristics appeared to be more associated with the appraisal of performance and handling of MPA after a performance than with the level of individual MPA before a performance.

To investigate the degree of correlation between situational MPA and dispositional MPA, a study with a new sample of *N* = 67 young amateur musicians from brass choirs was conducted (Spahn et al., 2023). Next to the PQM, the participants also completed the Kenny Music Performance Anxiety Inventory (K-MPAI) (Kenny, 2023). The K-MPAI showed moderate negative correlations (0.3 < *r* > 0.42) with all self-efficacy scales and with all other PQM scales after the performance. This confirmed the relationship between dispositional MPA and self-efficacy (Zelenak, 2024) even in a specific performance situation. However, the results revealed only a rather small correlation (*r* = 0.26) with the symptoms of MPA scale before the performance. This suggests that situational MPA before a performance was less associated with dispositional MPA, indicating that the level of anxiety directly before a performance is rather individual and depends more on situational factors than on the disposition of MPA. Regarding the previous classifications of MPA types (Spahn et al., 2021a), the study identified 75% of the young musicians being assigned to the positive first MPA type.

#### 2.2.4 Adaptation of the PQM in a mobile application

Due to the fact that every performance differs from the next, retest reliability is rather difficult to assess for the PQM questionnaire. However, certain aspects of the PQM may have individual stability and can provide important insights into one’s own approach to performances. Therefore, repeatedly completing the PQM in different performance situations would provide valuable details about individual stable and variable characteristics in handling MPA.

To make this possible, the PQM was implemented in the mobile application “Stage:Cool.” The PQM, the performance quality scale, and the questions on personal importance, concert difficulty, and performance difficulty were included in the mobile application. In addition, questions regarding ensemble and audience sizes were incorporated. The results of the PQM can be viewed immediately after completion.

The mobile application provides the ability to store the data from each performance on the device. It collects a database of performance details and the PQM results. The data can be used as a feedback tool or shared with the Freiburg Institute for Musicians’ Medicine (FIM) through an agreed data transfer. To date, 17 individual datasets have been transferred to the FIM, with a range between one and 14 performances. The data serves as a basis for discussion with a consultant from FIM if the performer wishes.

The mobile application of the PQM is available for Android and iOS devices (currently only in German).

## 3 Validation of the PQM

### 3.1 Validation sample

For the validation, two samples from previous studies and data from the mobile application were combined. From Spahn et al. (2021a), there were 530 participants, and from another study by Spahn et al. (2023), there were 58 participants with complete datasets of the PQM (no missing data) included. The first performance of the transmitted data from the 17 musicians was also included. The validation sample therefore contained *N* = 605 musicians, with 57% female and 43% male participants. The mean age was 30.6 years (SD = 14.1 years; min. 13 years; max. 74 years).

### 3.2 Statistics

The data were analysed using IBM SPSS Statistics (IBM Corp., Armonk, N. Y., USA, Version 30) and R (Version 4.5.2; R Core Team; https://www.R-project.org/). Descriptive statistics are reported with the mean and the standard deviation (SD) of the mean. Items were tested for univariate normality using the Shapiro–Wilk test, and at each time point of the performance, the items were tested for multivariate normality using the Mardia test. Pearson’s correlations were used for numeric variables, and Kendall’s tau correlations for ordinal variables. A multivariate analysis of variance (ANOVA) was performed to compare numeric variables. Main effects were reported using *F*- and *p*-values. For significant results, paired analyses were conducted using a one-sided *t*-test. The level of significance was set to *p* = 0.05.

The exploratory factor analysis (EFA) was performed in IBM SPSS and used the principal axis factoring method with Promax rotation with Kappa = 4 (Grieder and Steiner, 2022). To determine the number of factors, the Kaiser criterion, which considers only factors with an eigenvalue greater than 1, was used. For internal consistency, Cronbach’s *α* and McDonald’s *ω* were reported. In addition, the Kaiser–Meyer–Olkin measure of sampling adequacy (KMO) and Bartlett’s Test of Sphericity were conducted.

The confirmatory factor analysis (CFA) was performed in R (lavaan Package) using the Satorra–Bentler robust maximum likelihood (MLM) estimator. For the model statistics, the *χ*^2^ and the degrees of freedom (df) were assessed. According to APA standards, the comparative fit index (CFI), the Tucker–Lewis Index (TLI), the root mean square error of approximation (RMSEA) with its 90% confidence interval, and the standardized root mean square residual (SRMR) were reported. Common cut-offs for judging model fit were used according to the literature (Hu and Bentler, 1999; Schermelleh-Engel et al., 2003).

### 3.3 Results of the validation

#### 3.3.1 Descriptive analysis

A table with the mean value, skewness, and kurtosis of each PQM item can be found in the Supplementary material. In the literature, items are considered to have acceptable skewness if the value is between −2 and +2, and acceptable kurtosis if the value is between −7 and +7 (Byrne, 2013; Hair et al., 2013). Kline describes a severe skewness for values above +/−3 and severe kurtosis for values above +/−10 (Kline, 2016). The skewness of all items lay within the acceptable range of −2 to 2. Two-thirds of the items showed skewness levels between 1.1 and 1.8 or between −1.1 and −1.6. A slightly slanted distribution of the responses had been expected for some items; however, they remained within the tolerable range for subsequent analyses. The kurtosis values were also within acceptable limits. The highest value was about 3.2, which is in accordance with Kline’s threshold of 6.2. However, considering the large sample size, these values may indicate non-normality of the items.

To test for normal distribution, univariate and multivariate tests were performed. All items were found to have a non-normal distribution (Shapiro–Wilk tests <0.001), and at all three time points of the performance, the multivariate tests also revealed non-normal distributions (Mardia tests <0.001). Accordingly, it was assumed that the data were not normally distributed. As normality is a crucial requirement in CFA for using standard maximum likelihood (ML) estimation, performing CFA with non-normally distributed data requires robust estimation methods. The CFA was therefore conducted using the Satorra–Bentler robust maximum likelihood estimation.

Correlation tables between the items can be found in the Supplementary material. Two different types of correlation tables are provided. First, there are three tables showing the correlations between all items at each time point of the performance. As expected, the items within the PQM scales showed the highest correlations compared to the other items. These correlations underline the structural concept of the PQM identified by the factor analyses.

Some items had higher correlations with items outside their own scale. During the performance, item 16 (functional coping scale) showed relatively high correlations with items 14, 20, and 21 (self-efficacy scale). Nevertheless, these were not the highest correlations for item 16. After the performance, item 30 (functional coping scale) also correlated with items 25, 29, and 32 (self-efficacy scale).

Second, three additional tables list the correlations between items across the performance according to Table 1. They demonstrate the connections of the items at the three time points, i.e., before, during, and after the performance. The items within the scale intended to be connected across the performance showed mainly the highest correlations. This was primarily found for the items between before and during the performance. However, between before and after the performance, the item connections often did not show the highest correlations within the same scale. Especially for the symptoms of MPA scale, while the correlations between before and during the performance were of medium degree (>0.5), the correlations between before and after the performance decreased to a low degree (<0.3) but were still significant.

#### 3.3.2 Exploratory factor analyses

The exploratory factor analyses (EFAs) were performed for each time point of the performance separately (Table 2). For the time point before the performance, the EFA confirmed a three-factor structure with 46% explained variance, with very good KMO (0.811) and Bartlett test (*p* < 0.001) results. The internal reliabilities of the PQM scales were acceptable to good, ranging from Cronbach’s *α* = 0.67 to *α* = 0.78.

**Table 2:** Factor loadings (EFA) of the items in each PQM scale with reliability scores (Cronbach’s *α* and McDonald’s *ω*) for the scales and how Cronbach’s *α* change if the item was removed.

PQM scale		PQM Item	Factor 1	Factor 2	Factor 3	*α* when item not included
Before the performance	Functional coping (*α* = 0.680; *ω* = 0.689)	8	0.707			0.530
		4	0.701			0.544
		1	0.493			0.676
	Symptoms of MPA (*α* = 0.789, *ω* = 0.789)	10		0.839		0.689
		9		0.674		0.760
		2		0.667		0.775
		6		0.650		0.729
	Self-efficacy (*α* = 0.667, *ω* = 0.670)	7			0.777	0.526
		5			0.572	0.617
		3			0.523	0.591
		11			0.437	0.657
During the performance (forced three-factor analysis)	Functional coping (*α* = 0.777, *ω* = 0.778)	15	0.839			0.672
		16	0.467		0.320	0.717
		22	0.333			0.684
	Symptoms of MPA (*α* = 0.794, *ω* = 0.797)	13		0.806		0.743
		17		0.725		0.729
		19		0.712		0.728
		12		0.548		0.770
	Self-efficacy (*α* = 0.761, *ω* = 0.745)	21			0.829	0.653
		20			0.682	0.716
		14			0.668	0.702
		18			0.575	0.743
After the performance	Functional coping (*α* = 0.784, *ω* = 0.786)	23			0.680	0.727
		26			0.631	0.697
		30	0.324		0.475	0.698
	Symptoms of MPA (*α* = 0.770, *ω* = 0.768)	31		0.630		0.725
		33		0.623		0.743
		28		0.503		0.705
		24		0.498		0.684
	Self-efficacy (*α* = 0.787, *ω* = 0.787)	29	0.809			0.659
		25	0.721			0.704
		32	0.615			0.776

The EFA for the time point during the performance showed a two-factor structure with 47% explained variance and very good KMO (0.855) and Bartlett test (*p* < 0.001) results. Items originally included in the functional coping scale were found to be loaded together with items belonging to the self-efficacy scale on the same factor. This seemed to be mainly caused by item 16, which correlated with items related to the self-efficacy scale (see correlation tables in the Supplementary material). Since the correlations for item 16 were not the highest, the EFA was forced to perform with three factors. The result was a clear structure of the intended three dimensions. Item 16 showed a loading on factor 3, but it was smaller than on factor 1. The explained variance was 53%. The internal reliabilities were good, ranging from Cronbach’s *α* = 0.76 to 0.79.

In the analysis of the time point after the performance, item 27 had to be excluded from the EFA due to a low communality score (0.079). The EFA showed a three-factor structure with 54% explained variance and very good KMO (0.893) and Bartlett test (*p* < 0.001) results. The internal reliabilities were good, ranging from Cronbach’s *α* = 0.77 to *α* = 0.79.

With these results from the three-factor solutions, the factors were clearly assigned to the three scales: factor 1 = functional coping; factor 2 = symptoms of MPA; and factor 3 = self-efficacy. At the time point during the performance, item 16 was included in the functional coping scale.

The EFA of the seven items of the self-rated performance quality yielded a clear one-factor structure with 51% explained variance. All items had factor loadings larger than 0.59, and the internal consistency of the scale with all items was Cronbach’s *α* = 0.87 (*ω* = 0.87).

#### 3.3.3 Confirmatory factor analyses

Confirmatory factor analyses (CFAs) were also conducted for each time point separately. Item 27 was excluded at the time point after the performance. To test whether the proposed three-factor solutions of the EFA could also be reproduced in the CFA, competing CFA models with one, two, and three factors were conducted. The model fit parameters without any correlated residuals at the three time points of the performance can be found in the table in the Supplementary material. The fit indices showed that the three-factor solution had the best model fit among the competing CFA models. Therefore, the three-factor structure was applied to all three time points of the performance.

Some modification indices (mi) between item errors showed values above the threshold of mi > 10 (Brown, 2015), indicating correlated item residuals. For the time point before the performance, these were found for the symptoms of MPA scale between items 6 and 10 (mi = 19.9) and for the self-efficacy scale between items 5 and 7 (mi = 14.5) as well as items 3 and 7 (mi = 13.4). The statements in items 3 and 5 reflect excitement about the upcoming performance and are therefore closely related to item 7. Items 6 and 10 focus on the effects of MPA in terms of feeling impaired. These item pairs were conceptually similar, which may have caused the error correlations. To test whether the added correlated error of these item combinations increased the model fit, the models were compared (ANOVA). The analyses showed that adding the correlated errors significantly increased the model fit indices (*p* < 0.001).

During the performance, modification indices (mi) above 10 were found between items 14 and 18 (mi = 16.9), items 14 and 20 (mi = 22.3), and items 20 and 21 (mi = 22.8). All item pairs were in the self-efficacy scale. Items 14, 18, and 20 were similar to the same items found at the time point before the performance with high modification indices. Those items point conceptually in a similar direction. Item 21 considers inner strength and aligns with item 20. The comparison analysis (ANOVA) between these models showed significant (p < 0.001) increases in the model fit parameters with added correlated errors.

Two item combinations after the performances had high modification indices above 10. These were between items 24 and 28 (mi = 10.4) and items 31 and 33 (mi = 17.5). Both items 24 and 28 address the difficulty of believing that the performance was successful. Items 31 and 33 similarly focus on stress-inducing thinking. The analyses between the models (ANOVA) showed that the model fit parameters increased significantly (*p* < 0.001) with added correlated errors.

The correlated errors between the above-mentioned item combinations affected the degrees of freedom (df) in the model fit. The statistics of the model fits are listed in Table 3. The three-factor solutions of the PQM scales at each time point provided adequate model fits according to the ranges of common cut-offs (Hu and Bentler, 1999; Schermelleh-Engel et al., 2003; Brown, 2015; Kline, 2016). Diagrams of the CFA models at all time points of the performance, with standardized factor loadings, residual variances, and correlated errors, can be found in the Supplementary material.

**Table 3:** Model fit parameters of the CFA for the PQM scale structures in the three time points.

Time point	*χ* ^2^	df	CFI	TLI	RMSEA	LO 90%	Hi 90%	SRMR
Before the performance	143.1	38	0.933	0.903	0.072	0.060	0.085	0.065
During the performance	160.5	38	0.937	0.909	0.082	0.069	0.095	0.060
After the performance	80.5	30	0.968	0.953	0.064	0.047	0.081	0.039

Robust Fit Indices using R (lavaan, Satorra–Bentler estimator) of the three-factor solution including error correlations; CFI, comparative fit index; TLI, Tucker–Lewis Index; RMSEA, root mean square error of approximation reported with 90% confidence interval (low and high); SRMR, standardized root mean square residual.

#### 3.3.4 Correlations between the PQM scales

A correlation matrix between all PQM scales and the performance quality scale is listed in Table 4. The symptoms of MPA scales were negatively correlated with the other scales. At each time point, the highest correlations were found between the functional coping and self-efficacy scales. Across the time points, high correlations were observed between the self-efficacy scales. The highest correlation was found in the symptoms of MPA scale between the time points before and during the performance.

**Table 4:** Correlations (Pearson’s *r*) between the PQM scales.

PQM Scale	Before the performance			During the performance			After the performance		
	FC	SoMPA	SE	FC	SoMPA	SE	FC	SoMPA	SE
Before FC	1	−0.34**	0.43**	0.66**	−0.34**	0.48**	0.35**	−0.30**	0.36**
Before SoMPA	–	1	−0.15*	−0.39**	0.71**	−0.28**	−0.20*	0.33**	−0.25**
Before SE	–	–	1	0.33**	−0.13*	0.66**	0.58**	−0.36**	0.55**
During FC	–	–	–	1	−0.48**	0.56**	0.42**	−0.39**	0.41**
During SoMPA	–	–	–	–	1	−0.31**	−0.23*	0.38**	−0.26**
During SE	–	–	–	–	–	1	0.69**	−0.46**	0.63**
After FC	–	–	–	–	–	–	1	−0.60**	0.65**
After SoMPA	–	–	–	–	–	–	–	1	−0.46**
After SE	–	–	–	–	–	–	–	–	1
Performance quality	0.13	−0.16*	0.37**	0.29**	−0.13	0.46**	0.44**	−0.27**	0.36**

FC, functional coping; SoMPA, symptoms of MPA; SE, self-efficacy, *p < 0.05, **p < 0.01.

The performance quality scale correlated mainly with the self-efficacy scale across all time points. The functional coping scale did not correlate before the performance but did after the performance. The values of the symptoms of MPA scale before and during the performance did not correlate with the performance quality scale.

No significant correlations were found between the functional coping and self-efficacy scales and the age of the musicians. In the symptoms of MPA scale, there were low but significant negative correlations with age found before the performance (*r* = −0.15*) and during the performance (*r* = −0.18*), indicating that older musicians had slightly fewer symptoms of MPA.

#### 3.3.5 Comparison analyses

**Figure 1:**
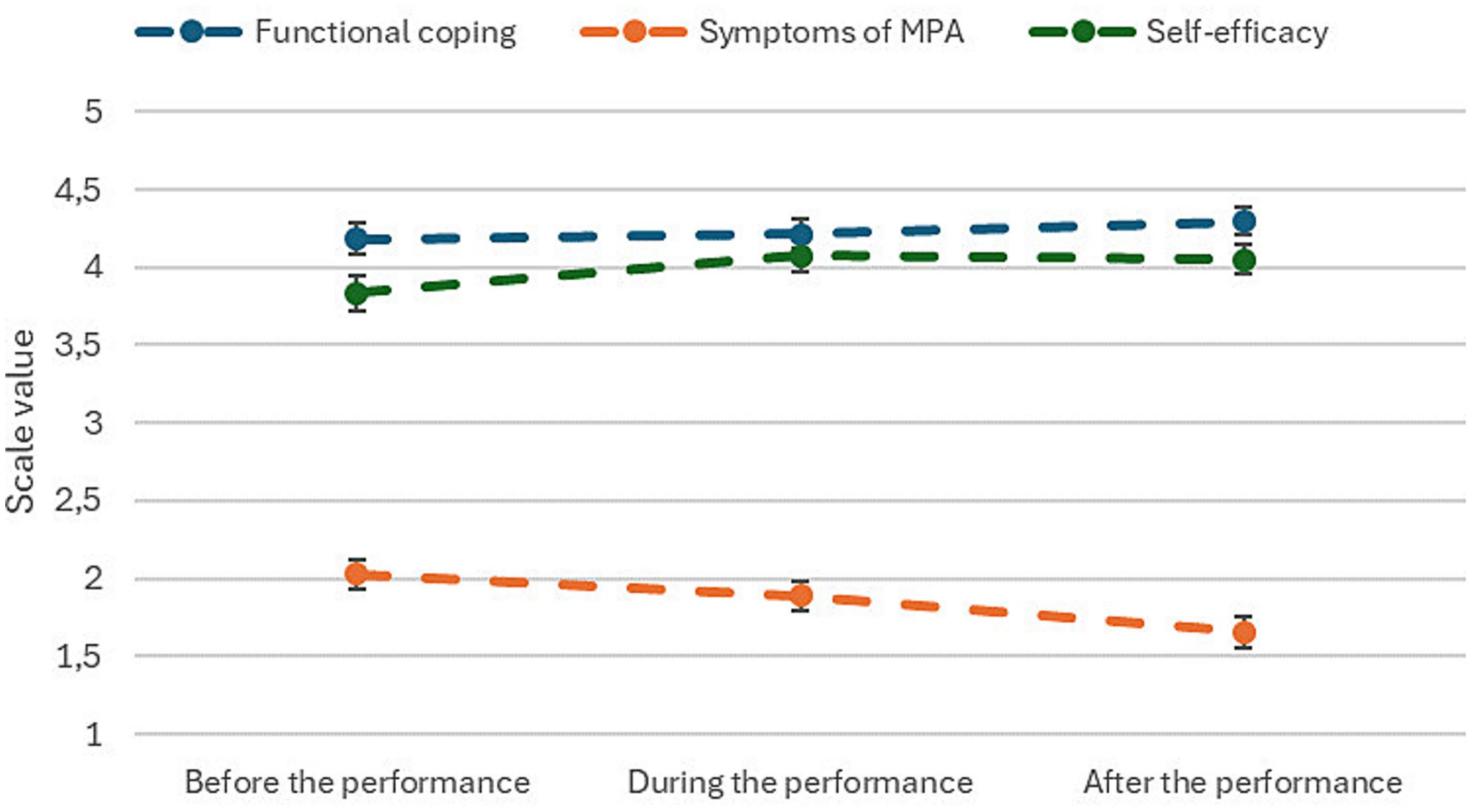
Mean values of the PQM scales in each time point with standard error (*n* = 605).

The mean values of the PQM scales can be seen in Figure 1. A table with the mean values can be found in the Supplementary material. Overall, the repeated measures analysis showed that the mean functional coping scale was consistently rather high (>4) and increased significantly across the performance [*F*(1,604) = 8.9, *p* = 0.003]. A paired analysis showed that there was no significant difference between the values before and during the performance [*t*(604) < 1], but a greater difference was observed between during and after the performance [*t*(604) = −2.5, *p* = 0.007]. In contrast, the symptoms of MPA scale steadily decreased across the performance [*F*(1,604) = 88.6, *p* < 0.001], with significant differences in the scale values between each time point.

In the self-efficacy scale, the main effect over the performance was significant [*F*(1,604) = 47.9, *p* < 0.001]. However, a paired analysis found that the value increased between before and during the performance [*t*(604) = −9.5, *p* < 0.001], but did not differ significantly between during and after the performance.

There were no significant differences in the mean values between genders in any of the PQM scales. However, as there are known gender differences in MPA (Sokoli et al., 2022), multiple group invariance model comparisons of the CFA models were performed at all three points in time of the performance for gender groups. The equalities of item factor loadings across gender were examined using a metric invariance model, and item intercepts across gender were examined using a scalar invariance model. The test statistics of the model comparisons can be found in the Supplementary material. According to Chen (2007), robust criteria for indicating measurement invariance are based on changes in RMSEA and CFI. Common thresholds include a ΔCFI <0.010 and a ΔRMSEA <0.015. In all multiple group model comparisons of the PQM the fit changes were below these thresholds indicating measurement invariance for all scales.

A multivariable analysis of variance was performed for the variables of personal importance, concert difficulty, and personal performance difficulty with all PQM scales and the performance quality scale. Only the main effects were calculated in the analyses. Table 5 lists the *p*-values for each main effect. The mean values for the PQM scales for the ratings of the three performance questions can be found in the Supplementary material.

**Table 5:** Statistics of the MANOVA for the comparison of the importance, concert difficulty and personal difficulty ratings of the musicians on all PQM scales.

PQM scale		Importance	Concert difficulty	Personal difficulty
		*F*(3,514)/*p*-value	*F*(3,514)/*p*-value	*F*(4,514)/*p*-value
Before the performance	Functional coping	2.01/0.087	0.79/0.498	**2.48/0.040**
	Symptoms of MPA	2.33/0.068	0.90/0.440	1.96/0.098
	Self-efficacy	**30.1/<0.001**	0.43/0.733	**10.1/<0.001**
During the performance	Functional coping	0.16/0.920	1.45/0.226	**4.57/0.001**
	Symptoms of MPA	1.25/0.248	0.99/0.394	**2.81/0.025**
	Self-efficacy	**14.2/<0.001**	0.72/0.543	**7.62/<0.001**
After the performance	Functional coping	**18.5/<0.001**	0.87/0.455	**3.99/0.003**
	Symptoms of MPA	**5.02/0.002**	0.62/0.604	**4.24/0.002**
	Self-efficacy	**17.6/<0.001**	0.54/0.654	**2.89/0.022**
Performance quality		**5.80/0.001**	0.66/0.572	**6.43/<0.001**

*F*-values and *p*-values are reported; in bold: significant values; tables with individual mean values of the PQM scales can be found in the Supplementary material.

The self-efficacy scale showed significant differences in the importance ratings at all time points. The more important the performance, the higher the values on the self-efficacy scale. The functional coping and symptoms of MPA scales also showed significant differences between importance ratings, but only after the performance. Higher importance ratings were associated with higher functional coping scores and lower symptoms of MPA scores.

The self-efficacy scale at each time point also showed significant differences in personal difficulty ratings. However, higher self-efficacy values were found when the difficulty was rated as just right (before and after the performance) or low (during the performance). Difficulty ratings of “too low” or “too high” were associated with lower self-efficacy values. Similarly, the functional coping scale showed significant differences within the difficulty ratings, with the highest values found for the low difficulty rating.

There was no difference in the symptoms of MPA scale before the performance according to the difficulty ratings. During the performance, the highest symptoms of MPA scale values were found for the high difficulty ratings. The highest symptoms of MPA scale values after the performance were found for the difficulty rating of “too high.”

The self-rated performance quality was also affected by how important and personally difficult the performance was. The higher the importance, the higher the quality rating. Conversely, the greater the personal difficulty, the lower the quality rating.

The ratings in concert difficulty, i.e., the comparison of this concert with other concerts, revealed no differences in the PQM scales.

### 3.4 Discussion of the validation

The EFA and the CFA confirmed the reliability of each scale and the scale structure at the three time points of the performance. While the EFA revealed a clear three-factor solution for the time points before and after the performance, a double loading of item 16 in the functional coping and the self-efficacy scales was found during the performance. Due to the correlations between this item and other items related to the same scale at that time point, and due to the theoretical basis of the clear content assignment of item 16 to the functional coping scale, the EFA was constrained to operate with three factors. Internal reliability was high for all scales. The internal reliabilities of the PQM scales in this larger validation sample confirmed the already reported reliabilities in other studies (Spahn et al., 2021a,b).

The CFA affirmed the structure with adequate model fit parameters. However, these model fits were slightly below the cut-offs for a good model fit. It is proposed that for good models, the CFI and TLI should be >0.95, RMSEA <0.05, and SRMR <0.08; and for acceptable model fits, CFI and TLI should be >0.90 and RMSEA <0.08 (Schermelleh-Engel et al., 2003; Brown, 2015; Kline, 2016; Goretzko et al., 2024). While in the models before and during the performance, the CFI indicated an acceptable fit, after the performance the CFI reached a level indicative of a good fit. In all three models, the TLI and the RMSEA showed acceptable fit parameters, and the SRMR was below the cut-off, indicating a good model fit.

Item 27 showed a very low factor loading in the EFA and was excluded from the analyses. It may be that switching off after a performance is a very individual characteristic that seems not to be related to the other dimensions of the PQM. Therefore, the use of the PQM should not include item 27.

The PQM was designed to assess performance-specific MPA aspects considering the time points before, during, and after the performance. The items were developed to conceptually point in the same direction across the performance. However, the items differ between the three time points due to the wording in retrospect and in the present tense. Therefore, the scales are not identical across the performance. Nevertheless, the scales are conceptually very similar and represent a connected relationship. This was also confirmed by the correlations at the scale and item level. Thus, a longitudinal interpretation across the performance is possible, but the structural comparison should be investigated in more detail.

The correlations between the individual PQM scales showed stability and independence among the scales and over the time points of the performance. The self-efficacy scale exhibited a relatively stable relationship with functional coping throughout the performance. Before the performance, the symptoms of MPA scale correlated very weakly with the self-efficacy scale. The correlations between both scales increased slightly during the performance. This indicates a rather individual level of MPA prior to a performance. Self-efficacy was found to have a predictive function for general MPA (González et al., 2018). For the individual level of situational MPA in a particular performance, it seems that other aspects related to the performance may have an additional effect. However, the moderating effect of self-efficacy on the management of situational MPA in a performance was already demonstrated in Spahn et al. (2021a). The degree of self-efficacy was associated with an increase or decrease in the symptoms of MPA scale throughout the performance.

Interestingly, self-efficacy during the performance was highly correlated with functional coping after the performance. Musicians who experienced strong self-efficacy during the performance also had higher positive coping values afterwards. This suggests some kind of “boost” for positive coping when one’s expectations were met during the performance.

The self-rating of the quality of the performance appeared to be less associated with the PQM scales before the performance. Neither functional coping nor the symptoms of MPA scale were related to the performance rating. Only the self-efficacy scale showed a correlation with the performance rating across the entire performance. Performance was rated better when self-efficacy was higher. This aligns with other findings that self-efficacy plays an important role in music performances (Zelenak, 2024).

The performance quality was also correlated with the functional coping scale after the performance. A positive experience of the performance, perhaps influenced by a self-efficacy “boost,” may have resulted in a more favourable self-judgment of the performance. However, musicians often tend to evaluate their performance with high levels of self-criticism and perfectionism, which can also negatively affect goal progress (Powers et al., 2011). Therefore, self-ratings of performance quality may be distorted by personal characteristics. Nevertheless, positive self-ratings can also serve as a form of self-affirmation and lead to a positive performance experience (Churchill et al., 2018). Of course, performances can also be affected by various factors such as playing errors or difficulties with musical expression. However, this study showed that higher self-efficacy was associated with a better performance rating. Considering the finding that self-efficacy was significantly negatively related to self-criticism (Powers et al., 2011), this suggests that self-rating reflects one’s experience of positive thinking. Self-assessment of one’s own performance could lead to a confrontation with one’s self-criticism. It may be interesting to compare self-ratings with the ratings of others. However, external ratings are often based on different judgment criteria than self-ratings. In the PQM, the self-rating of performance is used as an additional value for evaluating subjective management of situational MPA.

Contrary to the findings that women have a higher level of general MPA (Kenny, 2011; Sokoli et al., 2022), this study showed no differences between genders. Since the focus here was not on general MPA, but on how musicians deal with situational MPA in a particular performance, it implies gender-independent behavior. The multiple group invariance comparison showed that the models were equally valid for both genders. However, potential differences in answering behavior for situational MPA, especially in relation to corresponding differences in general MPA between genders, should be researched in more detail.

Regarding the importance of the performance for the musicians, the self-efficacy scale was positively correlated at all time points of the performance. The more important the performance was, the higher the self-efficacy scale. One might have expected that an important performance would be associated with nervousness and tension, leading to reduced self-efficacy and higher situational MPA. The results showed that this was not the case, and the musicians’ level of MPA before the performance was not influenced by the importance of the performance. On the contrary, the self-efficacy scale was even higher when the performance was deemed more important. It seems that the significance of the performance may lead to higher self-efficacy. Further studies should investigate whether the greater subjective significance of a performance leads to more intensive preparation and thus increases self-efficacy. Moreover, after the performance, the functional coping and self-efficacy scales remained elevated, and the performance was rated as better according to the higher significance of the performance.

In contrast, the personal difficulty of the performance showed a non-linear relationship with the PQM scales. Difficulty ratings that were too low or too high were associated with lower self-efficacy and functional coping scale values. With regard to the performance model of Yerkes and Dodson (1908) and its different shaping related to task difficulty (Twitchell et al., 2025), performances rated as too simple might not have activated useful coping strategies, whereas those rated as too difficult might have intimidated the musicians. Further research is needed on the relationship between stress models, MPA, and self-efficacy. However, the situational MPA before the performance was not influenced by the difficulty ratings. This scale was affected only after the performance. The connections between the performance questions and the handling of situational MPA can be confirmed by the MPA types found in a cluster analysis in Spahn et al. (2021a).

## 4 Case study with the mobile application of the PQM

One musician collected data from several performances using the mobile application of the PQM. This dataset was used in a case study to evaluate individual differences across performances. The case study showed that the use of the mobile application is practical and leads to the identification of potential characteristics in the handling of MPA across several performances.

This study was conducted as a proof of concept to test the practicability of the mobile application and to examine one person’s results from the PQM in different performance situations.

### 4.1 Method and design

The data from the mobile application of the PQM of one musician were used in a case study. At the time of the study, the musician was pursuing a Bachelor of Music at the University of Music in Freiburg. Over approximately 6 months, she collected 31 performances in the mobile application’s database. The data were transferred to the Freiburg Institute for Musicians’ Medicine for analysis.

In the analysis, mean values of all PQM scales were compared with the mean values of the validation sample using one-sided *t*-tests. To analyse possible changes in the PQM scales over time, Pearson correlations between the PQM scales and the dates of completion were calculated. Since the points in time were not equidistant, it was not possible to perform a time series analysis. The Durbin–Watson test was conducted to detect autocorrelation of the scales, where a value around 2 (between 1.5 and 2.5) indicates independent residuals.

### 4.2 Results of the mobile application study

**Figure 2:**
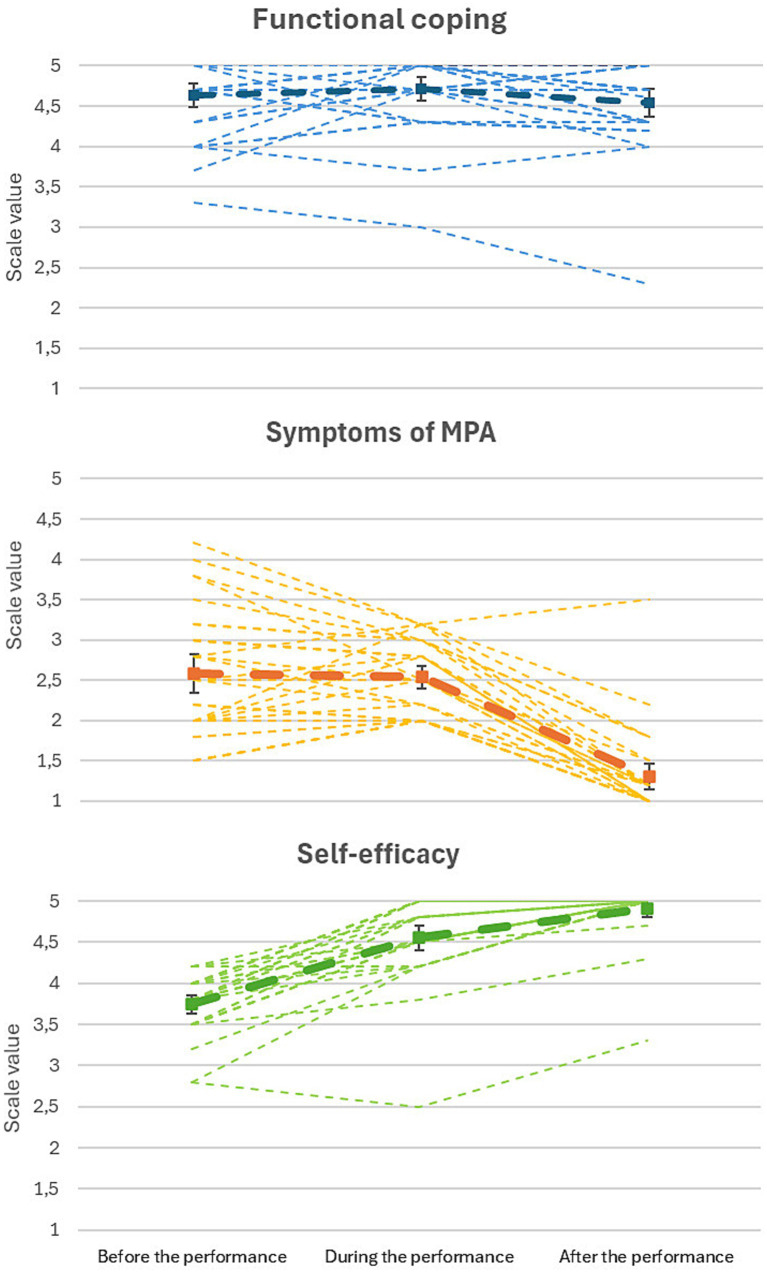
Mean values (bold line) with standard error of the PQM scales from the case study (*n* = 1) with the mobile application (the light lines are the individual performances, *n* = 31).

The mean values of the PQM scales are shown in Figure 2. The mean value of the functional coping scale was high (>4.5) across the performances, with no significant differences between the time points. The self-efficacy scale was similar to the mean value in the validation sample before the performances and increased significantly during the performance [*t*(30) = 11.1, *p* < 0.001] and after the performance [*t*(30) = 6.9, *p* < 0.001].

The symptoms of MPA scale before the performance was significantly higher than the mean value in the validation sample [*t*(30) = 3.5, *p* < 0.001] and remained at that level during the performance without significant difference. After the performance, the mean scale value reduced significantly [*t*(30) = 15.6, *p* < 0.001], falling below the mean value in the validation sample [*t*(30) = −4.1, *p* < 0.001].

Before the performance, the functional coping scale correlated with *r* = −0.59** with the symptoms of MPA scale, but not significantly with the self-efficacy scale (*r* = 0.33). During and after the performance, the functional coping and self-efficacy scales correlated at high levels (*r* > 0.8). After the performance, the symptoms of MPA scale also correlated with both other scales at high levels (*r* > −0.79).

All PQM scales showed no autocorrelation, with Durbin–Watson values between 1.8 and 2.4, except for the symptoms of MPA scale during the performance, which had a value of 2.6 indicating negative autocorrelation. This scale also had a significant negative correlation with the date of completion of the questionnaire (*r* = −0.41*), while the other scales showed no correlations. Over time, the symptoms of MPA during the performance decreased.

Regarding the number of co-musicians in the performance and the size of the audience, the highest correlation was found between the number of performers and the symptoms of MPA scale before the performance (*r* = −0.39*). The more persons performing, the lower the symptoms of MPA. The second highest correlation was between the size of the audience and the symptoms of MPA scale before the performance, with a correlation of *r* = −0.29, although it was slightly insignificant.

### 4.3 Discussion of the mobile application study

In a case study, the mobile application of the PQM was tested for its practicality and used to collect long-term data from the same person. The musician reported that filling out the PQM immediately after the performance using the mobile application was very easy and not disruptive. She also noted that she became faster with each completion and that it took very little time to complete the PQM in the mobile application. In addition, she was able to mentally put the performance behind her after completing it. Occasionally, out of curiosity, she liked to look at previous performances and compare her self-assessment against the data from the mobile application. The data were discussed individually with the musician in a feedback session.

The results of all PQM scales in the single subject study showed a similar range as in the validation study, with high functional coping, increasing self-efficacy, and decreasing symptoms of MPA. The functional coping scale before the performance was slightly higher than in the validation sample and did not increase over the time points. The average values reached a ceiling effect, i.e., they were not influenced by their MPA in terms of a positive assessment of performance.

The symptoms of MPA scale was at a rather high level before the performances and reduced during the performance. After the performance, it decreased drastically to a relatively low level. The individual performance values of the scale before the performance showed a high standard deviation of the mean, with SD = 0.8. After the performance, the standard deviation reduced to SD = 0.5. The results indicated that the musician generally had a relatively high degree of symptoms of MPA before the performance. After the performance, the symptoms of MPA scale reached a low level. In individual performances, it is notable that in some cases, the symptoms of MPA scale was low before the performance, increased during the performance, and decreased again after the performance. In other performances, the scale showed high values (>4) before the performance. In these cases, the scale value decreased steadily throughout the performance. The correlation with the timing of filling in the questionnaire also showed that the symptoms of MPA during the performance reduced steadily. This indicates that the musician decreased MPA in the performances over time. Whether this is due to increased experience or previous performances needs to be investigated in further studies.

The mean self-efficacy scale value before the performance was similar to the mean values in the validation sample. During and after the performance, the mean values increased drastically and reached a ceiling effect at the maximum of the scale. Interestingly, the individual performances showed that the musician almost always began performances with lower self-efficacy than she ended them. The correlations suggest that, for her, functional coping was more strongly associated with the symptoms of MPA than self-efficacy before the performance. It may be that training to increase self-efficacy before the performance could relate to the symptoms of MPA and reduce the large variation in that scale or even lower the mean value before the performance.

There was a correlation between the number of performers in the musician’s ensembles and the symptoms of MPA scale before the performance. The more performers who played alongside her, the lower the symptoms of MPA scale. In general, this confirms other studies on dispositional MPA, which have shown that when performing in ensembles, MPA is lower than in solo performances (Robson and Kenny, 2017). The musician reported that she also included auditions in the database. These are often performances in a small ensemble or even as a soloist in front of a small audience comprising a jury. In a feedback session, these performances should be given more consideration.

In Figure 2, there is one performance that stands out with low functional coping, low self-efficacy, and high symptoms of MPA. That was a particular audition where the musician accompanied a colleague and stated that it was a stressful event and she had little time to practice. This performance took place sometime between other performances. The following performances were not affected by this event and showed similar PQM scale values to those before that performance. The mobile app provides data to analyse individual behavior patterns for handling situational MPA and also to identify stable characteristics across performances.

## 5 Conclusion

In the present study, the Performance-related Questionnaire for Musicians (PQM) was validated as an instrument for assessing situational MPA at different time points during a performance, i.e., before, during, and after the performance. The PQM refers to a specific performance and must be completed directly after that performance. Filling out the questionnaire has proven to be very practical and easy to implement.

The questions of the PQM refer retrospectively to the time points immediately before and during the performance and concurrently to the time point after the performance. Three scales are measured for each of these time points: the symptoms of MPA scale, the functional coping scale, and the self-efficacy scale.

It has been shown that answering retrospectively the questions regarding the time point before the performance is valid (Biwer, 2015). It is not recommended to use the questionnaire in general surveys without reference to a particular performance. The results would then relate more to general dispositional MPA than to situational MPA. Furthermore, the PQM is an instrument that not only relates to a specific performance but also assesses the management of MPA over the course of that performance. The questionnaire thus serves as an important supplement to the established instruments that assess general MPA. Moreover, the PQM enables time-limited pre–post comparisons in intervention studies and offers certain opportunities for researching specific aspects of performance science. The PQM was developed to provide a tool for evaluating specific aspects of MPA with a clear reference to a particular performance, in order to facilitate and promote the investigation of those influencing factors.

The implementation of the PQM questionnaire in a mobile application has proven to be very useful. This has simplified the process of completing the questionnaire, and the option of saving performance situations in a database for later review has been well received. It should be noted that the mobile application is not intended for personal diagnosis, but rather as a feedback tool for individual use in understanding and managing one’s own MPA.

The results of the case study with the mobile application showed that individual characteristics can be derived from the stored data of multiple performances. This demonstrated that the proof of concept for using the PQM as a mobile application was successful. Therefore, it can be used not only in a scientific context but also for personal use and teaching purposes.

### 5.1 Limitations and future directions

Although the sample size for the PQM is already quite large, more specific music groups, such as amateur musicians, should be included. Factors influencing different manifestations of MPA in performance situations should also be investigated in more detail. Additionally, the relationship between situational MPA, as assessed by the PQM, and dispositional MPA should be further researched. For this purpose, future studies could also include the PQM in specific intervention studies that address individual management of MPA.

One question (item 27) in the current version of the PQM has been identified as unusable. This item had been excluded from the analyses. It would be advisable to check the stability of the PQM structure in a questionnaire without this item.

An interesting area for future research would be to incorporate external ratings or objective measures for judging performance quality. These measures could be used to investigate differences in relation to the PQM’s self-assessed performance ratings and the occurrence of situational MPA.

The mobile application of the PQM has proven useful. However, more data need to be collected from the mobile application in various performance situations. Further research is necessary to investigate the identification of individual characteristics in handling MPA across performances, as well as to explore the relationship between these characteristics and performance outcomes. To achieve this, the PQM should be provided in more languages, especially in the mobile application. The versions in English and Polish will soon be implemented.

## Data Availability

The raw data supporting the conclusions of this article will be made available by the authors, without undue reservation.
