# Supplementary material for: Development and validation of the German Performance-related Questionnaire for Musicians (PQM) for measuring situational music performance anxiety
Source: Front Psychol. 2026 Mar 3;17:1722181. doi: 10.3389/fpsyg.2026.1722181 (PMC12992244; doi:10.3389/fpsyg.2026.1722181)
Supplement: Supplementary file 1 [file Data_Sheet_1.pdf]

Supplementary Material

Validation and further developments of the Performance-related Questionnaire for Musicians (PQM) for measuring situational music performance anxiety

Claudia Spahn and Manfred Nusseck*

*** Correspondence:** Corresponding Author: Manfred.nusseck@uniklinik-freiburg.de

Content of the supplementary material:

1. Table with mean values of all PQM items and descriptive statistics
2. Table with mean values of the seven performance quality items
3. Correlation tables between the items in each time point of the performance
4. Correlation tables of the items of each PQM scale across the time points of the performance
5. Model fit parameters of competing CFA models
6. Visualizations of the CFA models
7. Table with mean values of all PQM scales and the performance quality scale
8. Multiple group invariance CFA model comparisons by gender
9. Table with mean values of all PQM scales and the performance quality scale by the ratings of the performance importance
10. Table with mean values of all PQM scales and the performance quality scale by the ratings of the concert difficulty
11. Table with mean values of all PQM scales and the performance quality scale by the ratings of the personal difficulty
12. The PQM questionnaire in German
13. The PQM questionnaire in English

1 PQM item statistics

Mean (between 1 and 5), standard deviation (SD) and skewness as well as kurtosis of all PQM items (N = 605, note: for kurtosis values according to Kline (2016) please add 3)

PQM	Mean	SD	Skewness	Kurtosis
Item01	4.11	.999	-1.124	.792
Item02	2.57	1.269	.301	-1.018
Item03	3.92	.917	-.751	.413
Item04	4.15	1.072	-1.454	1.627
Item05	3.57	1.267	-.622	-.625

Item06	1.79	1.090	1.307	.755
Item07	3.93	1.095	-1.050	.590
Item08	4.30	.935	-1.627	2.744
Item09	2.06	1.194	.973	-.032
Item10	1.75	.999	1.342	1.198
Item11	3.87	1.030	-.928	.522
Item12	1.80	1.034	1.249	.886
Item13	2.39	1.215	.457	-.806
Item14	4.13	.954	-1.192	1.220
Item15	4.11	1.054	-1.316	1.287
Item16	4.26	.851	-1.436	2.734
Item17	1.77	1.007	1.352	1.273
Item18	4.02	.943	-1.025	1.150
Item19	1.71	1.026	1.510	1.583
Item20	4.08	.959	-1.056	.874
Item21	4.02	.935	-.986	1.014
Item22	4.25	.919	-1.542	2.677
Item23	4.37	.851	-1.570	2.767
Item24	1.62	1.005	1.635	1.896
Item25	4.22	1.013	-1.303	1.192
Item26	4.40	.835	-1.546	2.471
Item27	3.80	1.268	-.767	-.534
Item28	1.65	1.033	1.543	1.470
Item29	4.22	.882	-1.209	1.460
Item30	4.09	.957	-1.062	.845
Item31	1.52	.919	1.894	3.170
Item32	3.67	1.110	-.582	-.313
Item33	1.89	1.066	1.130	.582

2 Performance quality item statistics

Mean (between 1 and 6), standard deviation (SD) and skewness as well as kurtosis of the seven self-rated performance quality items (N = 466, note: for kurtosis values according to Kline (2016) please add 3)

**Performance quality**	**Mean**	**SD**	**Skewness**	**Kurtosis**
Item01	4.33	.816	-.274	1.652
Item02	4.31	.916	-.250	.559
Item03	4.50	.890	-.177	-.069
Item04	4.68	.849	-.169	-.074
Item05	4.39	.893	-.152	.453
Item06	4.25	.995	-.353	.759

Item07	4.44	.758	.048	.934

3 Correlation tables between the items in each time point of the performance

3.1 Before the performance

	item01	item02	item03	item04	item05	item06	item07	item08	item09	item10	item11
item01	1	.046	.362**	.362**	.242**	-.101*	.293**	.375**	-.116**	-.116**	.299**
item02		1	-.166**	-.199**	.061	.465**	.086*	-.203**	.355**	.532**	-.040
item03			1	.323**	.331**	-.212**	.393**	.282**	-.248**	-.303**	.335**
item04				1	.188**	-.389**	.191**	.515**	-.265**	-.385**	.152**
item05					1	-.008	.462**	.155**	-.006	-.050	.187**
item06						1	-.039	-.304**	.470**	.577**	-.093*
item07							1	.209**	-.069	-.106**	.329**
item08								1	-.187**	-.341**	.208**
item09									1	.580**	-.182**
item10										1	-.141**
item11											1
	Functional coping scale										
	Symptoms of MPA										
	Self-efficacy scale										

3.2 During the performance

	item12	item13	item14	item15	item16	item17	item18	item19	item20	item21	item22
item12	1	.435**	-.213**	-.322**	-.293**	.458**	-.127**	.472**	-.326**	-.318**	-.303**
item13		1	.005	-.316**	-.150**	.535**	.044	.525**	-.232**	-.231**	-.329**
item14			1	.325**	.421**	-.108**	.520**	-.138**	.344**	.479**	.258**
item15				1	.531**	-.380**	.293**	-.389**	.309**	.355**	.564**
item16					1	-.253**	.281**	-.273**	.474**	.500**	.507**
item17						1	-.048	.544**	-.292**	-.270**	-.405**
item18							1	-.077	.295**	.370**	.164**
item19								1	-.259**	-.317**	-.379**
item20									1	.653**	.412**
item21										1	.482**
item22											1
	Functional coping scale										
	Symptoms of MPA										
	Self-efficacy scale										
	Correlations outside the own scale										

3.3 After the performance

	item23	item24	item25	item26	item28	item29	item30	item31	item32	item33
item23	1	-.436**	.407**	.536**	-.502**	.423**	.538**	-.349**	.348**	-.277**
item24		1	-.237**	-.485**	.591**	-.287**	-.388**	.459**	-.248**	.428**
item25			1	.434**	-.333**	.640**	.515**	-.366**	.493**	-.290**
item26				1	-.460**	.449**	.576**	-.389**	.447**	-.301**
item28					1	-.319**	-.379**	.420**	-.276**	.393**

item29						1	.564**	-.405**	.557**	-.277**
item30							1	-.364**	.503**	-.352**
item31								1	-.309**	.455**
item32									1	-.245**
item33										1
	Functional coping scale									
	Symptoms of MPA									
	Self-efficacy scale									
	Correlations outside the own scale									

4 Correlation tables of the items of each PQM scale across the time points of the performance

In gray: the intended relationship between the items according to Table 1 in the main manuscript.

4.1 Functional coping scale

		During the performance			After the performance		
		item15	item16	item22	item23	item26	item30
Before the performance	item01	.266**	.372**	.265**	.251**	.270**	.227**
	item04	.598**	.398**	.417**	.246**	.247**	.174**
	item08	.543**	.401**	.593**	.183**	.251**	.211**
During the performance	item15				.251**	.250**	.236**
	item16				.354**	.374**	.374**
	item22				.245**	.288**	.275**

4.2 Symptoms of MPA scale

		During the performance				After the performance			
		item12	item13	item17	item19	item24	item28	item31	item33
Before the performance	item02	.305**	.574**	.381**	.388**	.044	.039	.105**	.154**
	item06	.381**	.452**	.538**	.452**	.258**	.185**	.251**	.254**
	item09	.560**	.311**	.356**	.391**	.221**	.171**	.201**	.295**
	item10	.491**	.459**	.452**	.601**	.228**	.217**	.285**	.351**
During the performance	item12					.248**	.197**	.296**	.355**
	item13					.118**	.044	.159**	.204**
	item17					.266**	.202**	.301**	.312**
	item19					.300**	.218**	.259**	.313**

4.3 Self-efficacy scale

		During the performance				After the performance		
		item14	item18	item20	item21	item25	item29	item32
Before the performance	item03	.423**	.324**	.404**	.497**	.352**	.496**	.385**
	item05	.434**	.431**	.138**	.264**	.340**	.251**	.279**
	item07	.595**	.407**	.215**	.336**	.412**	.362**	.325**
	item11	.303**	.240**	.400**	.372**	.252**	.341**	.199**
	item14					.448**	.452**	.377**

During the performance	item18					.396**	.369**	.373**
	item20					.221**	.395**	.401**

5 Model fit parameters of competing CFA models

CFA was performed with one, two and three factors. The two and three factor models were created according to the findings of forced EFA. The table includes the robust fit indices with the Satorra-Bentler estimator. Item 16 was assigned to the Functional coping scale. After the performance, item 27 was excluded.

	χ^2	df	CFI	TLI	RMSEA	LO 90%	Hi 90%	SRMR
**One-factor**								
Before the performance	535.7	44	.618	.523	.159	.147	.171	.133
During the performance	518.3	44	.695	.619	.167	.154	.180	.114
After the performance	261.9	35	.838	.792	.134	.119	.150	.078
**Two-factors**								
Before the performance	291.7	43	.834	.788	.106	.095	.118	.088
During the performance	313.7	43	.843	.799	.121	.109	.134	.084
After the performance	155.9	34	.915	.888	.099	.083	.115	.062
**Three-factors**								
Before the performance	198.4	41	.899	.865	.085	.073	.097	.075
During the performance	233.7	41	.899	.864	.100	.087	.112	.069
After the performance	92.4	32	.961	.946	.069	.053	.085	.043

6 Visualisations of the CFA models

Abbreviations of the scales: FunCop: Functional coping, SympMPA: Symptoms of MPA, Selfefficacy: Self-efficacy. Item 16 was assigned to the Functional coping scale.

6.1 Before the performance

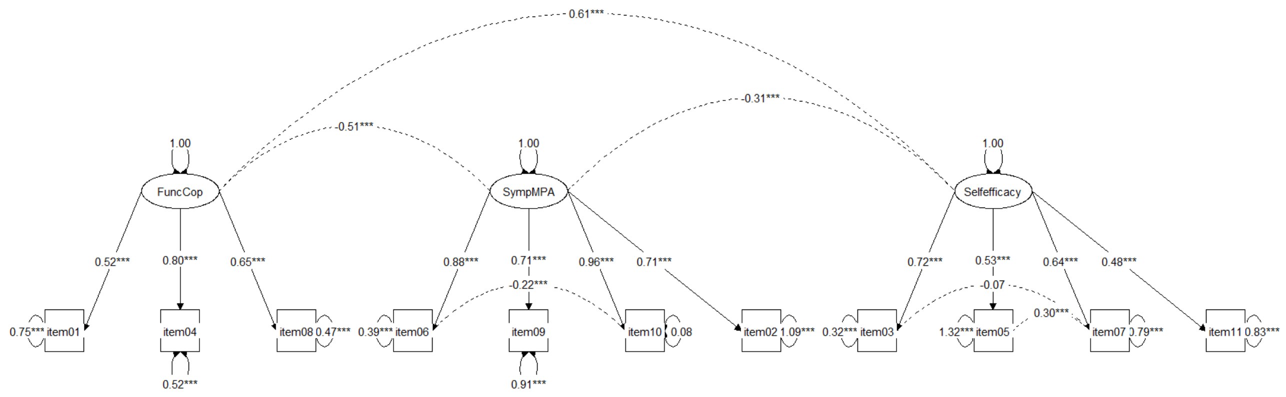

6.2 During the performance

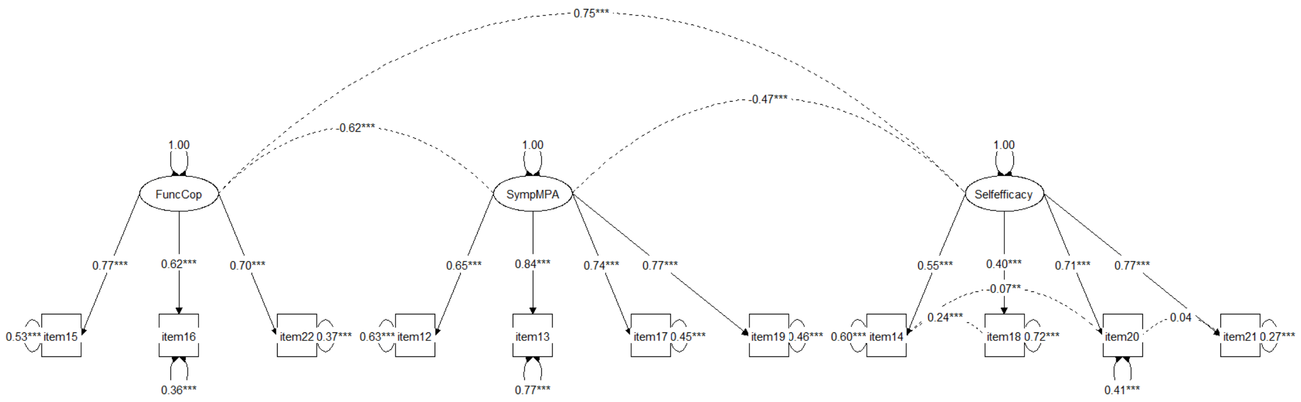

6.3 After the performance

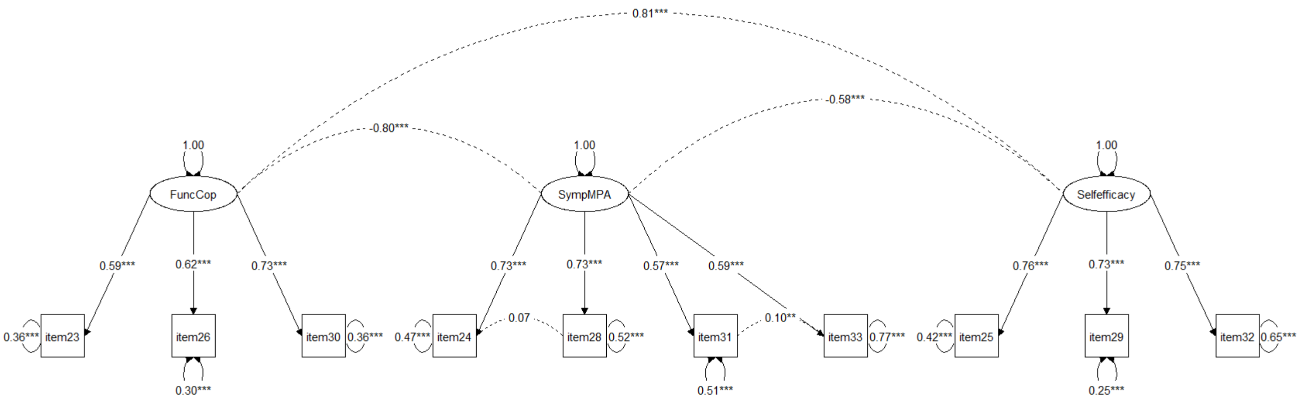

7 PQM scale statistics

Mean and standard deviation (SD) of the PQM scales and the performance quality scale. Item 16 was assigned to the Functional coping scale. After the performance, item 27 was excluded.

		Mean	N	SD
**Before the performance**	Functional coping	4.18	605	0.79
	Symptoms of MPA	2.04	605	0.89
	Self-efficacy	3.83	605	0.77
**During the performance**	Functional coping	4.20	605	0.79
	Symptoms of MPA	1.91	605	0.88
	Self-efficacy	4.07	605	0.72
**After the performance**	Functional coping	4.29	605	0.73

Symptoms of MPA	1.67	605	0.77
Self-efficacy	4.04	605	0.84
**Self-rated performance quality**	4.41	524	0.64

8 Multiple group invariance CFA model comparisons by gender

Item 16 was assigned to the Functional coping scale. After the performance, item 27 was excluded.

	$\chi^2/\Delta\chi^2$	df/ Δdf	CFI/ ΔCFI	RMSEA/ ΔRMSEA
**Before the performance**				
Configural	204.3	76	.932	.072
Metric	6.6	8	.001	.003
Scalar	7.1	8	.000	.003
**During the performance**				
Configural	240.3	76	.939	.079
Metric	4.5	8	.002	.005
Scalar	14.4	8	.002	.002
**After the performance**				
Configural	178.1	60	.963	.070
Metric	8.8	7	.001	.003
Scalar	21.5	7	.006	.001

9 Importance of the performance

Mean values (with standard deviation SD) of the PQM scales and the performance quality scale by the ratings of the personal importance of the performance. Item 16 was assigned to the Functional coping scale. After the performance, item 27 was excluded.

How important do you rate this performance for you personally?	Unimportant			Not that important			Important			Very important		
	M	N	SD	M	N	SD	M	N	SD	M	N	SD
Before, Functional coping	4,13	5	0,55	3,99	105	0,99	4,23	275	0,71	4,21	159	0,76
Before, Symptoms of MPA	1,65	5	0,74	1,75	105	0,83	2,08	275	0,84	2,26	159	0,92
Before, Self-efficacy	2,30	5	0,41	3,41	105	0,83	3,85	275	0,66	4,12	159	0,74
During, Functional coping	4,46	5	0,76	4,20	105	0,94	4,19	275	0,73	4,20	159	0,79
During, Symptoms of MPA	1,50	5	0,70	1,67	105	0,78	1,95	275	0,81	2,05	159	0,87
During, Self-efficacy	3,35	5	0,85	3,77	105	0,81	4,08	275	0,66	4,25	159	0,69
After, Functional coping	3,60	5	0,64	3,94	105	0,86	4,32	275	0,66	4,54	159	0,60

After, Symptoms of MPA	2,25	5	1,04	1,80	105	0,81	1,61	275	0,75	1,56	159	0,72
After, Self-efficacy	4,00	5	1,10	3,64	105	1,02	4,02	275	0,77	4,36	159	0,64
Performance quality	4,02	4	1,39	4,41	99	0,72	4,35	262	0,56	4,52	153	0,64

10 Concert difficulty of the performance

Mean values (with standard deviation SD) of the PQM scales and the performance quality scale by the ratings of the concert difficulty compared to other performances. Item 16 was assigned to the Functional coping scale. After the performance, item 27 was excluded.

**Compared to other performances was this performance…**	**Easy**			**Not that easy**			**Rather hard**			**Hard**		
	**M**	**N**	**SD**	**M**	**N**	**SD**	**M**	**N**	**SD**	**M**	**N**	**SD**
Before. Functional coping	4,14	133	0,97	4,26	202	0,72	4,15	156	0,75	4,05	48	0,72
Before. Symptoms of MPA	1,83	133	0,92	2,05	202	0,81	2,21	156	0,88	2,40	48	0,96
Before. Self-efficacy	3,78	133	0,85	3,94	202	0,69	3,78	156	0,75	3,61	48	0,88
During. Functional coping	4,26	133	0,95	4,19	202	0,78	4,12	156	0,71	4,28	48	0,64
During. Symptoms of MPA	1,69	133	0,85	1,94	202	0,76	2,08	156	0,89	2,04	48	0,83
During. Self-efficacy	4,04	133	0,82	4,16	202	0,63	4,00	156	0,73	3,95	48	0,73
After. Functional coping	4,25	133	0,75	4,39	202	0,67	4,30	156	0,70	4,13	48	0,86
After. Symptoms of MPA	1,65	133	0,83	1,62	202	0,71	1,66	156	0,71	1,95	48	1,00
After. Self-efficacy	4,01	133	0,94	4,13	202	0,81	4,02	156	0,75	3,93	48	0,91
Performance quality	4,53	119	0,74	4,39	197	0,58	4,38	150	0,58	4,30	48	0,74

11 Personal difficulty of the performance

Mean values (with standard deviation SD) of the PQM scales and the performance quality scale by the ratings of the personal difficulty of the performance. Item 16 was assigned to the Functional coping scale. After the performance, item 27 was excluded.

For me personally was the performance requirements…	Too low			Low			Just right			High			Too high		
	M	N	SD	M	N	SD	M	N	SD	M	N	SD	M	N	SD
Before. Functional coping	4,09	15	1,04	4,37	78	0,67	4,20	369	0,80	3,98	71	0,69	3,91	11	0,90
Before. Symptoms of MPA	1,43	15	0,53	1,71	78	0,78	2,11	369	0,87	2,33	71	0,93	2,25	11	1,19
Before. Self-efficacy	3,63	15	0,81	3,79	78	0,78	3,96	369	0,72	3,48	71	0,73	2,75	11	0,87
During. Functional coping	4,56	15	0,69	4,52	78	0,64	4,16	369	0,82	4,07	71	0,61	3,88	11	0,97
During. Symptoms of MPA	1,25	15	0,45	1,66	78	0,83	1,95	369	0,80	2,17	71	0,88	2,20	11	1,20
During. Self-efficacy	4,15	15	0,70	4,17	78	0,66	4,12	369	0,71	3,76	71	0,74	3,43	11	0,93
After. Functional coping	4,00	15	0,70	4,38	78	0,60	4,37	369	0,70	4,10	71	0,79	3,64	11	1,04
After. Symptoms of MPA	1,67	15	0,65	1,55	78	0,70	1,63	369	0,74	1,86	71	0,84	2,43	11	1,18
After. Self-efficacy	4,16	15	0,94	3,98	78	0,93	4,12	369	0,79	3,80	71	0,82	3,52	11	1,22
Performance quality	4,72	14	0,60	4,59	68	0,71	4,43	358	0,62	4,16	68	0,52	3,99	11	0,82

12 The PQM questionnaire in German

The following tables contain the German version of the PQM and the performance quality questionnaire.

	**Wenige Minuten vor dem Auftritt…**	1 (**trifft nicht zu**)	2	3	4	5 (**trifft sehr zu**)
**1**	… konnte ich mich auf die bevorstehende Aufgabe konzentrieren.	☐	☐	☐	☐	☐
**2**	… spürte ich Anzeichen von Aufregung in meinem Körper.	☐	☐	☐	☐	☐
**3**	... fühlte ich mich innerlich stark für den bevorstehenden Auftritt.	☐	☐	☐	☐	☐
**4**	... ist es mir gelungen, mich nicht von meiner Aufregung aus der Ruhe bringen zu lassen.	☐	☐	☐	☐	☐
**5**	… habe ich mir vorgestellt, dass sich das Publikum über meine Darbietung freuen wird.	☐	☐	☐	☐	☐
**6**	... habe ich mich durch körperliche Reaktionen meiner Aufregung beeinträchtigt gefühlt.	☐	☐	☐	☐	☐
**7**	… hatte ich Lust aufzutreten und zu zeigen, was ich kann.	☐	☐	☐	☐	☐
**8**	… habe ich meine Aufregung unter Kontrolle bekommen.	☐	☐	☐	☐	☐
**9**	… dachte ich daran, was alles schief gehen könnte.	☐	☐	☐	☐	☐
**10**	… war ich durch meine Aufregung verunsichert.	☐	☐	☐	☐	☐
**11**	… fühlte ich mich gut vorbereitet.	☐	☐	☐	☐	☐

	**Während des Auftritts…**	1	2	3	4	5
**12**	… habe ich ständig daran gedacht, dass etwas nicht klappen könnte.	☐	☐	☐	☐	☐
**13**	… spürte ich Aufregung in meinem Körper.	☐	☐	☐	☐	☐
**14**	… hatte ich Spaß und wollte zeigen, was ich kann.	☐	☐	☐	☐	☐
**15**	... ist es mir gelungen, mich nicht von meiner Aufregung aus der Ruhe bringen zu lassen.	☐	☐	☐	☐	☐
**16**	… konnte ich mich auf mein Spiel/ auf meine sängerische Darbietung konzentrieren.	☐	☐	☐	☐	☐
**17**	... habe ich mich durch körperliche Reaktionen meiner Aufregung beeinträchtigt gefühlt.	☐	☐	☐	☐	☐
**18**	... hatte ich das Gefühl, dass meine Darbietung beim Publikum ankommt.	☐	☐	☐	☐	☐
**19**	… war ich durch meine Aufregung verunsichert.	☐	☐	☐	☐	☐
**20**	… wusste ich genau, was ich tat.	☐	☐	☐	☐	☐
**21**	... fühlte ich mich innerlich stark.	☐	☐	☐	☐	☐
**22**	… habe ich meine Aufregung unter Kontrolle bekommen.	☐	☐	☐	☐	☐

**Jetzt, nach dem Auftritt…**		1	2	3	4	5
**23**	… denke ich, dass meine Darbietung beim Publikum gut angekommen ist.	☐	☐	☐	☐	☐
**24**	… fällt es mir schwer zu meiner Darbietung zu stehen.	☐	☐	☐	☐	☐
**25**	… freue ich mich schon auf meinen nächsten Auftritt.	☐	☐	☐	☐	☐
**26**	… stehe ich zu meiner Darbietung.	☐	☐	☐	☐	☐
**27**	… kann ich abschalten.	☐	☐	☐	☐	☐
**28**	... fällt es mir schwer zu glauben, dass meine Darbietung dem Publikum gefallen hat.	☐	☐	☐	☐	☐
**29**	… fühle ich mich innerlich stark für den nächsten Auftritt.	☐	☐	☐	☐	☐
**30**	… freue ich mich über meine Leistung.	☐	☐	☐	☐	☐
**31**	… fühle ich mich gestresst/unwohl.	☐	☐	☐	☐	☐
**32**	… fühle ich mich darin bestätigt, dass mir Auftreten liegt.	☐	☐	☐	☐	☐
**33**	… denke ich hauptsächlich daran, was nicht so gut geklappt hat.	☐	☐	☐	☐	☐

**Meine persönliche musikalische Leistung beurteile ich …**		**schlecht**<br>1	**eher schlecht**<br>2	**weniger gut**<br>3	**gut**<br>4	**sehr gut**<br>5	**Ausge-zeichnet**<br>6
**1**	… in der dynamischen Gestaltung als	☐	☐	☐	☐	☐	☐
**2**	… in der rhythmischen Präzision als	☐	☐	☐	☐	☐	☐
**3**	… in der klanglichen Gestaltung als	☐	☐	☐	☐	☐	☐
**4**	… im musikalischen Ausdruck als	☐	☐	☐	☐	☐	☐
**5**	… in der Phrasierung als	☐	☐	☐	☐	☐	☐
**6**	… in der Intonation als	☐	☐	☐	☐	☐	☐
**7**	… insgesamt als	☐	☐	☐	☐	☐	☐

13 The PQM questionnaire in English

The following tables contain an English version of the PQM and the performance quality questionnaire. Please note that this is a double translated version, but at the time of this publication, it was not validated.

	**A few minutes before the performance...**	1 (Not true at all)	2	3	4	5 (Very true)
**1**	… I could concentrate on the work at hand.	☐	☐	☐	☐	☐
**2**	… I could sense signs of agitation in my body.	☐	☐	☐	☐	☐
**3**	… I felt inner strength for the performance I was about to do.	☐	☐	☐	☐	☐
**4**	… I managed to control my agitation and stay calm.	☐	☐	☐	☐	☐
**5**	… I could imagine the audience enjoying my performance.	☐	☐	☐	☐	☐
**6**	… I felt limited or disabled due to the way my body reacted to my agitation.	☐	☐	☐	☐	☐
**7**	… I was looking forward to going on stage and showing what I could do.	☐	☐	☐	☐	☐
**8**	… I could control my agitation.	☐	☐	☐	☐	☐
**9**	… I thought about all the things that could go wrong.	☐	☐	☐	☐	☐
**10**	… my agitation made me unsure.	☐	☐	☐	☐	☐
**11**	… I felt well prepared.	☐	☐	☐	☐	☐
	**During the performance…**	1	2	3	4	5
**12**	… I couldn't stop thinking about what might go wrong.	☐	☐	☐	☐	☐
**13**	… I could feel the agitation in my body.	☐	☐	☐	☐	☐
**14**	… I had fun and wanted to show what I could do.	☐	☐	☐	☐	☐
**15**	… I managed to control my agitation and stay calm.	☐	☐	☐	☐	☐
**16**	… I could concentrate on my musical performance.	☐	☐	☐	☐	☐
**17**	… I felt limited or disabled due to the way my body reacted to my agitation.	☐	☐	☐	☐	☐
**18**	… I had the feeling that my performance came across well.	☐	☐	☐	☐	☐
**19**	… my agitation made me unsure.	☐	☐	☐	☐	☐
**20**	… I knew exactly what I was doing.	☐	☐	☐	☐	☐
**21**	… I felt inner strength.	☐	☐	☐	☐	☐
**22**	… I could control my agitation.	☐	☐	☐	☐	☐

Now, after the performance...

		1	2	3	4	5
**23**	… I think my performance came across well.	☐	☐	☐	☐	☐
**24**	… it's hard to accept my performance as it was.	☐	☐	☐	☐	☐
**25**	… I'm looking forward to my next performance.	☐	☐	☐	☐	☐
**26**	… I accept my performance.	☐	☐	☐	☐	☐
**27**	… I can switch off.	☐	☐	☐	☐	☐
**28**	… it's hard to imagine that the audience liked my performance.	☐	☐	☐	☐	☐
**29**	… I feel inner strength for the next performance.	☐	☐	☐	☐	☐
**30**	… I am happy about my achievement.	☐	☐	☐	☐	☐
**31**	… I feel stressed, unhappy.	☐	☐	☐	☐	☐
**32**	… this performance proves that performing suits me.	☐	☐	☐	☐	☐
**33**	… I'm mainly thinking about what didn't work.	☐	☐	☐	☐	☐

When considering the musical quality of my performance, I rate...

		**terrible**	**pretty bad**	**less good**	**good**	**very good**	**excellent**
		1	2	3	4	5	6
**1**	… the dynamic shaping as …	☐	☐	☐	☐	☐	☐
**2**	… the rhythmic precision as …	☐	☐	☐	☐	☐	☐
**3**	… the shaping of sound as …	☐	☐	☐	☐	☐	☐
**4**	… the musical expression as …	☐	☐	☐	☐	☐	☐
**5**	… the phrasing as …	☐	☐	☐	☐	☐	☐
**6**	… the intonation as …	☐	☐	☐	☐	☐	☐
**7**	… it in general as…	☐	☐	☐	☐	☐	☐
